# Rare Earth Element Transfer from Soil to Navel Orange Pulp (*Citrus sinensis* Osbeck cv. Newhall) and the Effects on Internal Fruit Quality

**DOI:** 10.1371/journal.pone.0120618

**Published:** 2015-03-25

**Authors:** Jinjin Cheng, Changfeng Ding, Xiaogang Li, Taolin Zhang, Xingxiang Wang

**Affiliations:** 1 Key Laboratory of Soil Environment and Pollution Remediation, Institute of Soil Science, Chinese Academy of Sciences, Nanjing, 210008, P R China; 2 University of the Chinese Academy of Sciences, Beijing, 100049, P R China; 3 Jiangxi Key Laboratory of Ecological Research of Red Soil, Ecological Experimental Station of Red Soil, Chinese Academy of Sciences, Yingtan, 335211, P R China; Old Dominion Univ., UNITED STATES

## Abstract

The effects of soil rare earth element (REE) on navel orange quality and safety in rare earth ore areas have gained great attention. This study investigated the transfer characteristics of REE from soil to navel orange pulp (*Citrus sinensis* Osbeck cv. Newhall) and examined the effects of soil REE on internal fruit quality in Xinfeng County, Jiangxi province, China. Path analysis showed that soil REE, pH, cation exchange capacity (CEC), and Fe oxide (Fe_ox_) significantly affected pulp REE concentrations. A Freundlich-type prediction model for pulp REE was established: log[REE_pulp_] = -1.036 + 0.272 log[REE_soil_] - 0.056 pH - 0.360 log[CEC] + 0.370 log[Fe_ox_] (n = 114, *R^2^* = 0.60). From the prediction model, it was inferred that even when soil REE and Fe_ox_ were as high as 1038 mg kg^-1^ and 96.4 g kg^-1^, respectively, and pH and CEC were as low as 3.75 and 5.08 cmol kg^-1^, respectively, pulp REE concentrations were much lower than the food limit standard. Additionally, soil REE levels were significantly correlated with selected fruit quality indicators, including titratable acidity (*r* = 0.52, P < 0.01), total soluble solids (*r* = 0.48, P < 0.01) and vitamin C (*r* = 0.56, P < 0.01). Generally, under routine methods of water and fertilization management, the cultivation of navel oranges in rare earth ore areas of south China with soil REE ranging from 38.6 to 546 mg kg^-1^ had improved in internal fruit quality.

## Introduction

The rare earth element (REE) comprise a group of 16 elements with very similar chemical and physical properties, including the lanthanides (Z = 57–71) and the element yttrium (Y, Z = 39) [1,2]. The lanthanides include lanthanum (La), cerium (Ce), praseodymium (Pr), neodymium (Nd), promethium (Pm), samarium (Sm), europium (Eu), gadolinium (Gd), terbium (Tb), dysprosium (Dy), holmium (Ho), erbium (Er), thulium (Tm), ytterbium (Yb), and lutetium (Lu). Among the lanthanides, the element Pm does not occur naturally on earth. REE are not as rare as the name implies. Their average abundance in the Earth’s crust is approximately 0.015%, which matches that of copper, lead and zinc [3]. Due to the small differences in their physical and chemical properties and their ionic radii, REE are generally divided into two groups: light REE (LREE) and heavy REE (HREE). La, Ce, Pr, Nd, Sm and Eu belong to the LREE; Gd, Tb, Dy, Ho, Er, Tm, Yb, Lu and Y constitute the HREE [1]. Fractionation of LREE and HREE often occurs during their transfer in soil-plant systems [4,5].

With the application of REE fertilizers, positive effects on the growth, yield and quality of numerous crops (including grains, vegetables and fruits) have been observed for pot or field experiments in many countries, including the United States, the United Kingdom and China [1,6]. However, REE in agricultural environments transfer to agricultural products through plant uptake, resulting in REE intake by humans, who are higher up the food chain [7]. Additionally, there is evidence that excessive exposure to REE is detrimental to human health [8,9]. Therefore, to ensure that REE levels in food are safe and to protect consumers against excessive exposure to REE in food, it is important to study the characteristics of REE transfer from soils to agricultural products and the major factors controlling that process. Much attention has been focused on the major controlling factors of REE transfer from soils to grains or vegetables [10,11]. These factors include soil REE content, pH, organic carbon (OC) content, cation exchange capacity (CEC), and clay content (< 0.002 mm) [10–12]. However, although these influencing factors are often interrelated, only simple correlation analyses were adopted in previous studies, making it difficult to determine how each factor individually contributes to the processes of REE transfer. Path analysis is one way to consider the complex variable relationships and can partition correlations into direct and indirect effects. Therefore, path analysis is a more comprehensive method than correlation analysis for processing complex variable relationships. Path analysis has been applied in the environmental and agricultural sciences to reveal, for example, the influences of soil properties on the adsorption of heavy metals [13] and the uptake of soil heavy metals by plants [14].

In addition, establishing empirical regression models is also an important method for determining whether the REE in crop production comply with the food safety limit. Numerous prediction models for the uptake of heavy metals by grains and vegetables have been developed [15–18]. These prediction models can be applied to back-calculate the threshold values for metals based on food quality standards [15,17] or to classify a particular soil as suitable or unsuitable for food production [18]. However, the number of prediction models for REE is limited, and most are established for the transfer of REE from soil to specific vegetables [10].

Researchers tend to establish prediction models based on the total content of heavy metals (exogenous metal salts) in the soil to meet the soil quality standards because most standards are based on total soil heavy metal content [14]. Since REE concentrations in plants usually do not depend on total endogenous REE concentrations (determined by strong acid digestion or alkali fusion) in their substrate soil [19,20], establishing regression models based on total soil REE concentrations does not seem feasible. The pseudo-total REE content using aqua regia (3:1, v/v, HCl to HNO_3_) digestion has been widely accepted in the environmental sciences as providing a good estimate of the maximal element availability for plant uptake [21], and it has been reported that the soil REE in aqua regia extracts are correlated with plant uptake [22]. Furthermore, the International Organization for Standardization (ISO) has standardized the aqua regia digestion procedure (DIN ISO 11466–1997 [23]), and the European Community Bureau of Reference has certified several soil samples for aqua regia extraction. Therefore, establishing a prediction model based on aqua regia extractable REE content is theoretically and practically feasible.

Ion-adsorption rare earth ore, also called deposit in weathered crust of granitic body, has a typical characteristic that 80%-90% of REE in the ore exist as adsorbed ions. It is widely distributed in Jiangxi, Fujian, Guangdong and Hunan provinces of south China. Because of the geological background and mining activities, soils in these regions are rich of REE. Summing up survey data from researchers, total soil REE concentrations in these regions ranged from 93.8 to 1038 mg kg^-1^, with an average of 220 mg kg^-1^ [24–26]. However, high soil REE levels have brought about food safety issues. It has been reported that REE contents in husked rice and some types of vegetables grown in rare earth ore areas of south China exceeded the food safety limit [26,27]. Navel oranges (*Citrus sinensis* L. Osbeck) are widely cultivated in south China. However, little research has been conducted on REE levels in navel orange pulp. Besides, effects of soil REE on internal fruit quality of navel oranges remain unclear. Xinfeng County of Jiangxi province, which is located in south China, is a representative ion-adsorption rare earth ore area and also an important location for navel orange production with a cultivated area of 13 000 ha and an output of 150 000 t in 2011. Therefore, the present study uses Xinfeng County as an example to (1) investigate the distribution and fractionation characteristics of REE in soil and navel orange pulp; (2) reveal major controlling factors and establish prediction models for REE transfer from soil to navel orange pulp; and (3) explore the relationship between soil REE content and internal fruit quality indicators, including titratable acidity (TA), total soluble solids (TSS) and vitamin C (Vc).

## Materials and Methods

### Ethics statement

The present study was carried out on collective-owned lands, and the owners of the navel orange orchards gave us permission to conduct the study on these sites. The field studies did not involve endangered or protected species.

### Study area

This study was performed in Xinfeng County, Jiangxi province, in south China (25°02′-25°23′ N, 114°39′-115°14′ E), which is one of the most famous navel-orange-growing areas in China. This area is situated in a subtropical, humid monsoon climate zone that is characterized by a warm climate, abundant sunshine and plentiful rainfall. The mean annual temperature is 19.6°C, and the ≥ 10.0°C accumulated temperature is 6882°C; the mean annual insolation time is 1596.8 h; the frostless season is 298 d; and the mean annual precipitation is 1510 mm. The soils of navel orange orchards primarily include red soil derived from granite (CST: Ali-Udic Cambosols; WRB: Umbic Acrisols), red soil derived from quartzite (CST: Ali-Udic Cambosols; WRB: Umbic Acrisols), red soil derived from argillaceous rock (CST: Argic-Udic Ferrosols; WRB: Petroplinthic Acrisols), calcareous purple soil derived from purple gravel rock (CST: Calcaric Purpli-Udic Cambosols; WRB: Haplic Cambisols) and acidic purple soil derived from purple gravel rock (CST: Dystric Purpli-Udic Cambosols; WRB: Haplic Cambisols). The major navel orange cultivar in Xinfeng County is the Newhall navel orange (*Citrus sinensis* Osbeck cv. Newhall), and the planting area of the Newhall navel orange accounts for 88% of the total navel orange planting area.

### Routine methods of water and fertilization management in navel orange orchards

Navel orange trees need to be fertilized four times per year. For each tree, the following types and amounts of fertilizers were used: 1) before spring sprouting, 0.15–0.5 kg urea and 0.15–0.5 kg compound fertilizer (N:P_2_O_5_:K_2_O = 15:15:15); 2) during the stage of autumn sprouting, 3–5 kg peanut cake fertilizer, 0.2–0.3 kg urea and 0.25–0.5 kg K_2_SO_4_; 3) after the fruit was picked, 0.25–0.5 kg compound fertilizer; 4) during the winter, 2–3 kg peanut cake fertilizer, 0.5 kg compound fertilizer and 1–1.5 kg calcium-magnesia phosphate fertilizer. If drought occurred during the blossom period and the fruit development period, irrigation was needed to maintain the soil moisture content in root zone at approximately 25% (gravity content) or 60% of field capacity.

### Soil and fruit sampling

**Fig 1:**
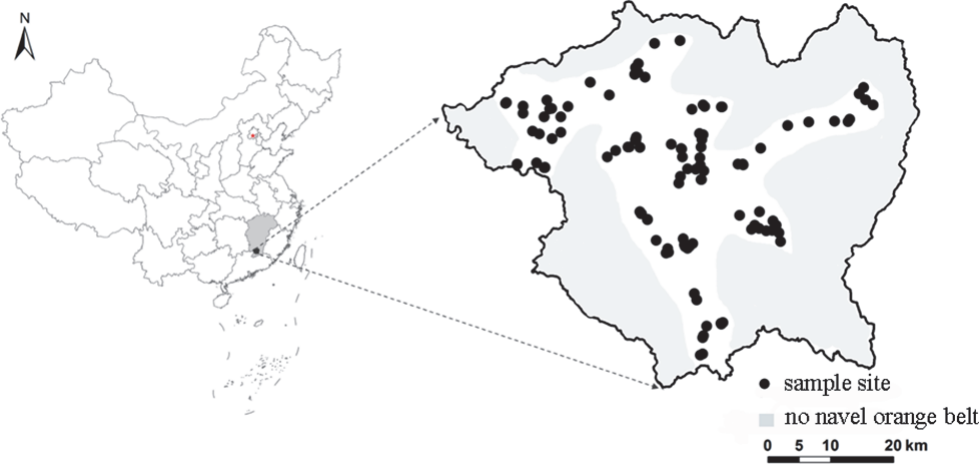
The locations of the soil sampling sites in Xinfeng County.

Based on the planting scale, the soil type and the age of the navel orange trees, 114 Newhall navel orange orchards (8–12 years old, *Citrus sinensis* Osbeck cv. Newhall) were chosen as monitoring sites for the study area (Fig. 1). The water and fertilization management of these orchards followed routine methods and were relatively consistent. In each orchard, five trees were selected as experimental units. Soil samples were collected at 0–50 cm from the drip line of navel orange trees during harvest in late November 2011. Fruit samples were taken from the trees in the areas where the soil samples were collected. The fruits were uniform in color and size and were taken from both the internal and external parts of the crown in four directions to compose a representative fruit sample for each orchard. At the same time, root and leaf samples were also collected for other research.

### Soil and fruit analysis

All soil samples were air-dried, ground, and sieved for analyses of the basic physical and chemical properties. The soil pH (1:2.5 soil-to-water ratio), OC content (K_2_CrO_4_-H_2_SO_4_ oil-bath heating), CEC (1 M CH_3_COONH_4_ leaching method at pH 7.0), particle size (hydrometer method), and Fe oxide content (extracted by HF-HNO_3_-HClO_4_) were analyzed according to the routine analytical methods of agricultural chemistry in soil [28]. Selected physical and chemical properties of the soils are shown in Table 1. Soil REE were extracted from finely ground soil material (< 150 μm) using the aqua regia digestion method according to DIN ISO 11466–1997 [23].

**Table 1:** Descriptive statistics of measured soil properties in 114 navel orange orchards.

	Unit	Minimum	Maximum	Mean	S.D.	CV (%)
pH		3.75	8.23	5.06	0.81	16
OC	g kg^−1^	3.17	17.3	7.37	2.75	37
CEC	cmol kg^−1^	5.08	35.8	10.3	4.38	42
clay	%	6.76	48.4	23.2	7.76	33
Fe_ox_	g kg^−1^	15.8	96.4	43.3	15.7	36

Fruit samples were brought to the laboratory immediately after harvesting. The fruit was washed with tap water, scrubbed gently in deionized water using a nylon brush to remove any superficial contamination, and peeled. The pulp of each navel orange was divided into two parts. For the determination of internal fruit quality, one part was homogenized using a Retsch grinder (GM 200, RETSCH, Germany); for the determination of REE content, the second part was dried first at 105°C for 30 min, then at 75°C in an oven until it was completely dry. It was then ground finely enough to pass through a 0.25 mm sieve. At the same time, the water content of the pulp was recorded. The pulp sample used for the determination of REE was digested with a mixture of HNO_3_ and HClO_4_ [10].

The internal fruit quality was determined immediately after the pulp was homogenized. Three representative internal fruit quality parameters for navel oranges were selected: titratable acidity (TA), total soluble solids (TSS) and vitamin C (Vc) [29]; the TA and TSS are indicators of the flavor, and the Vc is a reflection of the nutritive value. The TA was determined using 0.01 mol L^-1^ NaOH with phenolphthalein as an indicator and expressed as the percentage of malic acid. The TSS was determined by a hand refractometer and expressed as a percentage, and the Vc was determined using 2,6-Dichlorophenolindophenol via the visual titrimetric method and expressed as mg ascorbic acid per 100 g fresh weight [30].

The REE concentrations in the soil and plant digestion solutions were determined by inductively coupled plasma-mass spectrometry (ICP-MS, Agilent 7700X, USA) following a modified EPA Draft Method 1638 (USEPA 1996 [31]). The detection limit for REE was 0.01 mg kg^-1^ for soil samples and 0.001 mg kg^-1^ for plant samples, and the precision was better than ± 5%. A certified reference soil material (GBW07407, National Research Center for Certified Reference Materials, China), with a total REE concentration of 265.17 ± 26.51 mg kg^-1^, and a certified reference plant material (citrus leaf; GBW10020, National Research Center for Certified Reference Materials, China), with a total REE concentration of 6.55 ± 1.19 mg kg^-1^, were used to ensure the precision of the analytical procedure. The recovery ratios of the REE in the reference soil and plant ranged from 86% to 95% and 93% to 109%, respectively, throughout the procedure. The reference soil (GBW07407) was produced to measure the total content of elements, which explains why the recovery ratio of the aqua regia soluble REE content in the reference soil was slightly low. Nevertheless, this reference soil was used because it had similar physical and chemical properties to the soil samples in the present study, and there was no certified reference soil material for the aqua regia soluble REE content.

### Data analysis

The transfer factor (TF) is used to evaluate the transfer potential of REE from the soil to the plant, which is defined as the ratio of REE content in the plant to the REE content in the soil:

$${\text{TF}}_{\text{REE}}=\frac{{\text{REE}}_{\text{pulp}}}{{\text{REE}}_{\text{soil}}}$$ (1)

$${\text{TF}}_{\text{LREE}}=\frac{{\text{LREE}}_{\text{pulp}}}{{\text{LREE}}_{\text{soil}}}$$ (2)

$${\text{TF}}_{\text{HREE}}=\frac{{\text{HREE}}_{\text{pulp}}}{{\text{HREE}}_{\text{soil}}}$$ (3)

where REE_soil_, LREE_soil_ and HREE_soil_ are the concentrations of REE, LREE and HREE in the soil and REE_pulp_, LREE_pulp_ and HREE_pulp_ are the concentration of REE, LREE and HREE in the pulp.

**Fig 2:**
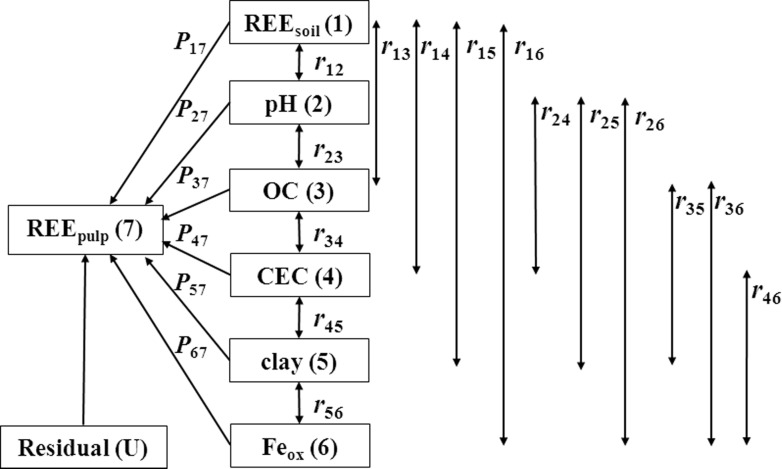
The path analysis diagram for the relationship between REE content in navel orange pulp and soil REE or soil properties. Single-headed arrows represent the direct effects (*P*_ij_), and double-headed arrows indicate the coefficients of the correlations between soil REE and the soil properties (*r*_ij_). Subscript designations for soil REE, the soil properties, and the REE content in the navel orange pulp are identified numerically as follows: (1) REE content in soil; (2) soil pH; (3) organic carbon (OC); (4) cation exchange capacity (CEC); (5) clay; (6) Fe oxide (Fe_ox_); (7) REE content in the pulp.

Path analysis (PA) was applied to reveal the influences of soil REE and soil properties on the uptake of REE by navel orange pulp, and it was performed separately for REE_pulp_, LREE_pulp_ and HREE_pulp_. Single-headed arrows represent direct effects of soil REE and soil properties on REE content navel orange pulp, and double-headed arrows indicate the coefficients of correlations between soil REE and soil properties (Fig. 2). The direct and indirect effects in the PA were obtained by multiple regression and simple correlation analyses. The correlation between REE content in navel orange pulp and soil REE or a given soil property is the sum of the direct and indirect coefficients:

$$r_{17}=P_{17}+r_{12}P_{27}+r_{13}P_{37}+r_{14}P_{47}+r_{15}P_{57}+r_{16}P_{67}$$ (4)

$$r_{27}=r_{12}P_{17}+P_{27}+r_{23}P_{37}+r_{24}P_{47}+r_{25}P_{57}+r_{26}P_{67}$$ (5)

$$r_{37}=r_{13}P_{17}+r_{2}{}_{3}P_{27}+P_{37}+r_{34}P_{47}+r_{35}P_{57}+r_{36}P_{67}$$ (6)

$$r_{47}=r_{14}P_{17}+r_{24}P_{27}+r_{34}P_{37}+P_{47}+r_{45}P_{57}+r_{46}P_{67}$$ (7)

$$r_{57}=r_{15}P_{17}+r_{25}P_{27}+r_{35}P_{37}+r_{4}{}_{5}P_{47}+P_{57}+r_{56}P_{67}$$ (8)

$$r_{67}=r_{16}P_{17}+r_{2}{}_{6}P_{27}+r_{3}{}_{6}P_{37}+r_{4}{}_{6}P_{47}+r_{5}{}_{6}P_{57}+P_{67}$$ (9)

where *r*_ij_ is the simple correlation coefficient between REE content in navel orange pulp and soil REE or a given soil property, *P*_ij_ is the direct effect, and *r*_ij_ *P*_ij_ is the indirect effect.

An uncorrelated residue (*U*) that represents the unexplained part of an observed variable in the path model was calculated using the following equation:

$$U=\sqrt{1-R^{2}}$$ (10)

where *R* ^2^ is the coefficient of determination of the multiple regressions between REE content in navel orange pulp and soil REE or soil properties.

A stepwise multiple linear regression (SMLR) analysis was used to derive the prediction models with pulp REE content as the dependent variable and soil REE content and soil properties as the independent variables. Using an SMLR methodology, only those factors that actually affect pulp REE content can enter the regression equation. The prediction models were established separately for the REE_pulp_, LREE_pulp_ and HREE_pulp_.

PA and SMLR were conducted using the statistical package SPSS 18.0. The method for variance of analysis was F-test, P < 0.05. Graphing was performed in Sigma Plot 11.0. All data (except for pH) were log-transformed prior to analysis due to their non-normal distributions.

## Results

### Content of REE in soil and navel orange pulp

The range and mean of the total content for each REE in soil and in navel orange pulp are listed in Table 2. The concentrations of total REE varied considerably, from 38.6 to 546 mg kg^-1^, with an average of 138 mg kg^-1^. The highest concentrations were observed for Ce, which varied from 13.9 mg kg^-1^ to 220 mg kg^-1^ and accounted for 41% of the total REE. With increasing atomic numbers, concentrations of the REE in the soil decreased in the following order: Ce > La > Nd > Y > Pr > Sm > Gd > Dy > Er > Yb > Eu > Tb > Ho > Tm > Lu. The distribution of the REE obeyed the Oddo-Harkins rule: the even-numbered REE were more abundant than their adjacent odd-numbered REE. The LREE accounted for 85% of the total REE, whereas the HREE were relatively rare.

**Table 2:** Contents of REE in soil and navel orange pulp.

	Soil (mg kg^−1^)				Pulp (mg kg^−1^)			
	Min	Max	Mean	S.D.	Min	Max	Mean	S.D.
La	6.19	153	25.1	19.8	0.015	0.353	0.086	0.062
Ce	13.9	220	57.0	35. 2	0.044	0.383	0.137	0.083
Pr	1.63	37.7	5.81	4.61	0.003	0.026	0.010	0.006
Nd	7.39	157	24.6	19.3	0.015	0.121	0.047	0.023
Sm	1.15	22.1	4.43	3.22	0.002	0.015	0.006	0.003
Eu	0.165	3.22	0.755	0.582	—^a^	0.007	0.002	0.001
Gd	0.672	14.8	3.31	2.58	0.001	0.028	0.006	0.004
Tb	0.087	1.75	0.416	0.346	—	0.003	0.001	0.001
Dy	0.437	9.20	2.14	1.93	0.001	0.020	0.004	0.003
Ho	0.078	1.78	0.397	0.388	—	0.003	0.001	0.001
Er	0.194	5.20	1.08	1.12	0.001	0.013	0.003	0.002
Tm	0.024	0.758	0.149	0.163	—	0.001	0.000	0.000
Yb	0.128	4.89	0.930	1.05	—	0.008	0.002	0.001
Lu	0.017	0.703	0.127	0.149	—	0.001	0.000	0.000
Y	2.26	57.9	11.5	12.0	0.008	0.118	0.036	0.024
LREE	31.6	456	118	73.4	0.089	0.728	0.288	0.148
HREE	4.13	90.7	20.1	19.6	0.012	0.180	0.054	0.034
REE	38.6	546	138	88.1	0.106	0.828	0.341	0.169

^a^ Below the detection limit.

The concentration of REE in navel orange pulp was very low, ranging from 0.106 to 0.829 mg kg^-1^ dry weight, with an average of 0.341 mg kg^-1^ dry weight. In general, individual REE partitioning exhibited the following order: Ce > La > Nd > Y > Pr > Gd > Sm > Dy > Er > Yb > Eu > Ho > Tb > Tm > Lu. The LREE accounted for approximately 84% of the total REE content, with Ce forming the largest share of the REE. The mean water content of the navel orange pulp was 87%. When converting the REE into rare earth oxide (REO) on the basis of fresh pulp weight, the total content of REO in the navel orange pulp was approximately 0.016–0.125 mg kg^-1^ fresh weight, with an average of 0.052 mg kg^-1^ fresh weight, which is far below the standard food safety limit (0.7 mg kg^-1^ fresh weight) stipulated by the national food and health regulations of China (MHPRC, 2005 [32]).

**Fig 3:**
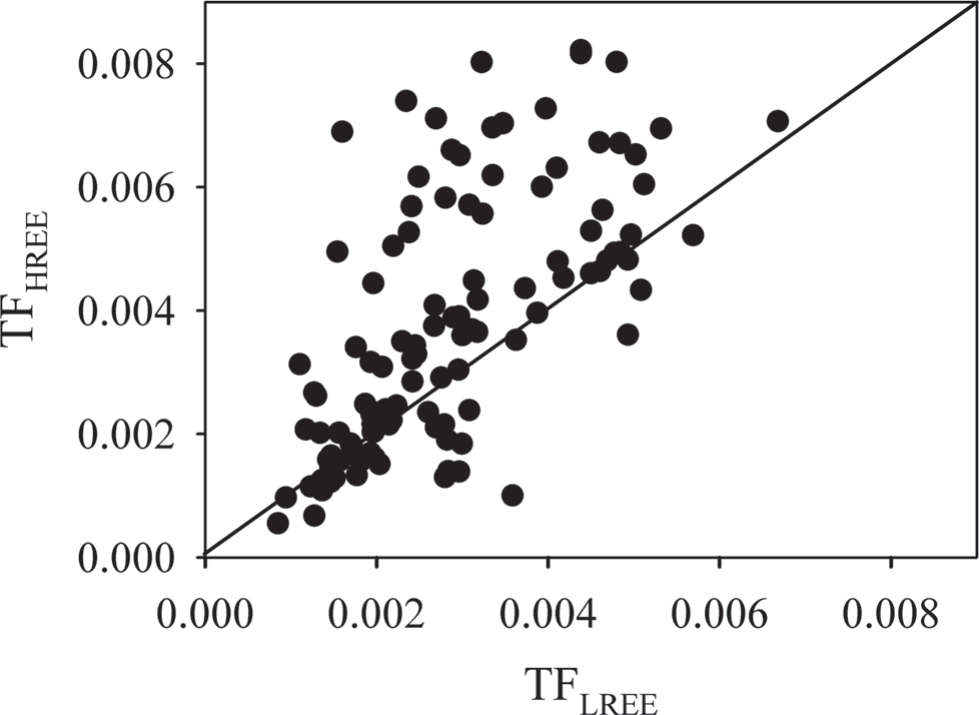
Transfer factors of LREE and HREE in the soil-navel orange system.

The TF_REE_ in the pulp ranged from 0.001 to 0.007, averaging 0.003. The fractionation between the LREE and HREE was very obvious (Fig. 3). Enrichment of HREE relative to the LREE in the navel orange pulp was detected. The ratio TF_HREE_/TF_LREE_ reflect the HREE enrichment, and most of the TF_HREE_/TF_LREE_ values in the pulp were higher than 1, indicating that the navel orange pulp had a greater ability to accumulate the HREE versus LREE.

### Major factors affecting REE uptake by navel orange pulp from soil

the pulp REE content The simple correlations between REE_pulp_ and REE_soil_ (*r* = 0.67, P < 0.01), pH (*r* = -0.55, P < 0.01), CEC (*r* = -0.44, P < 0.01), and Fe_ox_ (*r* = 0.56, P < 0.01) were significant (Table 3). Path analysis (PA) was applied to separate these simple correlations into direct effects and indirect effects. The uncorrelated residual value (*U*) was 0.63, and the coefficient of determination (*R* ^2^) was 0.61, indicating that the PA explained 61% of the variation in the REE_pulp_ (Table 3). The PA identified significant direct effects of the REE_soil_ (*P*_17_ = 0.34, P < 0.001), pH (*P*_27_ = -0.21, P < 0.01), CEC (*P*_47_ = -0.26, P < 0.001), and Fe_ox_ (*P*_67_ = 0.25, P < 0.01) on the REE_pulp_, whereas the direct effects of the OC (*P*_37_) and clay (*P*_57_) were not significant. Furthermore, the PA revealed that the indirect effects of the REE_soil_ through Fe_ox_ (*r*_16_ *P*_67_ = 0.14), pH through REE_soil_ (*r*_21_ *P*_17_ = -0.17), CEC through REE_soil_ (*r*_41_ *P*_17_ = -0.12), and Fe_ox_ through REE_soil_ (*r*_61_ *P*_17_ = 0.20) also have important effects on the pulp REE content.

**Table 3:** Direct effects (diagonal, italics) and indirect effects (off diagonal) of soil properties and soil REE on pulp REE.

	R_soil_ ^a^	pH	OC	CEC	clay	Fe_ox_	*r*	*R* ^2^	*U*
LREE_pulp_									
LREE_soil_	*0*.*36* ***	0.10	0.00	0.08	0.00	0.14	0.68**	0.60	0.63
pH	−0.18	−*0*.*19* **	0.01	−0.08	0.00	−0.09	−0.54**		
OC	0.01	−0.02	*0*.*06*	−0.02	0.02	0.02	0.08		
CEC	−0.12	−0.06	0.00	−*0.24* ***	0.02	−0.01	−0.41**		
clay	0.00	0.00	0.02	−0.07	*0.06*	0.07	0.08		
Fe_ox_	0.21	0.07	0.01	0.01	0.02	*0*.*24* **	0.57**		
HREE_pulp_									
HREE_soil_	*0*.*45* ***	0.06	0.01	0.07	0.00	0.05	0.65**	0.54	0.68
pH	−0.17	−*0*.*17* *	0.00	−0.07	0.00	−0.06	−0.48**		
OC	−0.09	−0.02	*−0*.*04*	−0.02	0.00	0.02	−0.14		
CEC	−0.14	−0.06	0.00	−*0*.*22* **	0.00	−0.01	−0.43**		
clay	−0.14	0.00	−0.01	−0.06	*0.00*	0.04	−0.17		
Fe_ox_	0.15	0.06	0.00	0.01	0.00	*0.17* *	0.38**		
REE_pulp_									
REE_soil_	*0*.*34* ***	0.10	0.00	0.09	0.00	0.14	0.67**	0.61	0.63
pH	−0.17	−*0*.*21* **	0.00	−0.08	0.00	−0.09	−0.55**		
OC	0.00	−0.02	*0*.*05*	−0.02	0.01	0.02	0.04		
CEC	−0.12	−0.07	0.00	−*0*.*26* ***	0.01	−0.01	−0.44**		
clay	−0.02	0.00	0.02	−0.07	*0.05*	0.07	0.03		
Fe_ox_	0.20	0.08	0.00	0.01	0.01	*0.25* **	0.56**		

^a^ Represents LREE_soil_, HREE_soil_ or REE_soil_

* Significant at P<0.05

** Significant at P<0.01

*** Significant at P<0.001.

The major factors affecting the transfer of LREE and HREE from soil to navel orange pulp are similar to those affecting the REE, except for the coefficient difference (Table 3).

### Prediction models for REE transfer from soil to navel orange pulp

To predict REE transfer from soil to the navel orange pulp, the Freundlich-type function is often used:

$${\text{C}}_{\text{plant}}=10^{\text{a}}{\text{C}}_{\text{soil}}{}^{\text{b}}\;\text{or}\;{\text{logC}}_{\text{plant}}=\text{a}+\text{b}\;{\text{logC}}_{\text{soil}}$$ (11)

where C_plant_ is the REE concentration in navel orange pulp, C_soil_ is the REE concentration in soil, and a and b are constants. A Freundlich-type function can be extended using soil properties such as pH, OC, CEC, and clay content, and the log-transformed Freundlich-type equation is commonly preferred.

Table 4 shows the prediction models for the LREE, HREE and total REE. The SMLR analysis identified LREE_soil_, pH, CEC and Fe_ox_ as factors that best explain the variability in LREE_pulp_ (*R* ^2^ = 0.60, P < 0.001). Similarly, the combination of HREE_soil_, pH, CEC and Fe_ox_ best explained the variability in HREE_pulp_ (*R* ^2^ = 0.53, P < 0.001). Approximately 60% (P < 0.001) of the variability in the REE_pulp_ was explained by REE_soil_, pH, CEC and Fe_ox_.

**Table 4:** Stepwise multiple linear regression equations.

Regression equations	n	*R* ^2^	P	RMSE	
log[LREE_pulp_] = −1.214 + 0.296 log[LREE_soil_] − 0.050 pH − 0.326 log[CEC] + 0.376 log[Fe_ox_]	114	0.59	<0.000	0.14	(12)
log[HREE_pulp_] = −1.499 + 0.346 log[HREE_soil_] − 0.056 pH − 0.389 log[CEC] + 0.260 log[Fe_ox_]	114	0.53	<0.000	0.18	(13)
log [REE_pulp_] = −1.036 + 0.272 log[REE_soil_] − 0.056 pH − 0.360 log[CEC] + 0.370 log[Fe_ox_]	114	0.60	<0.000	0.14	(14)

**Fig 4:**
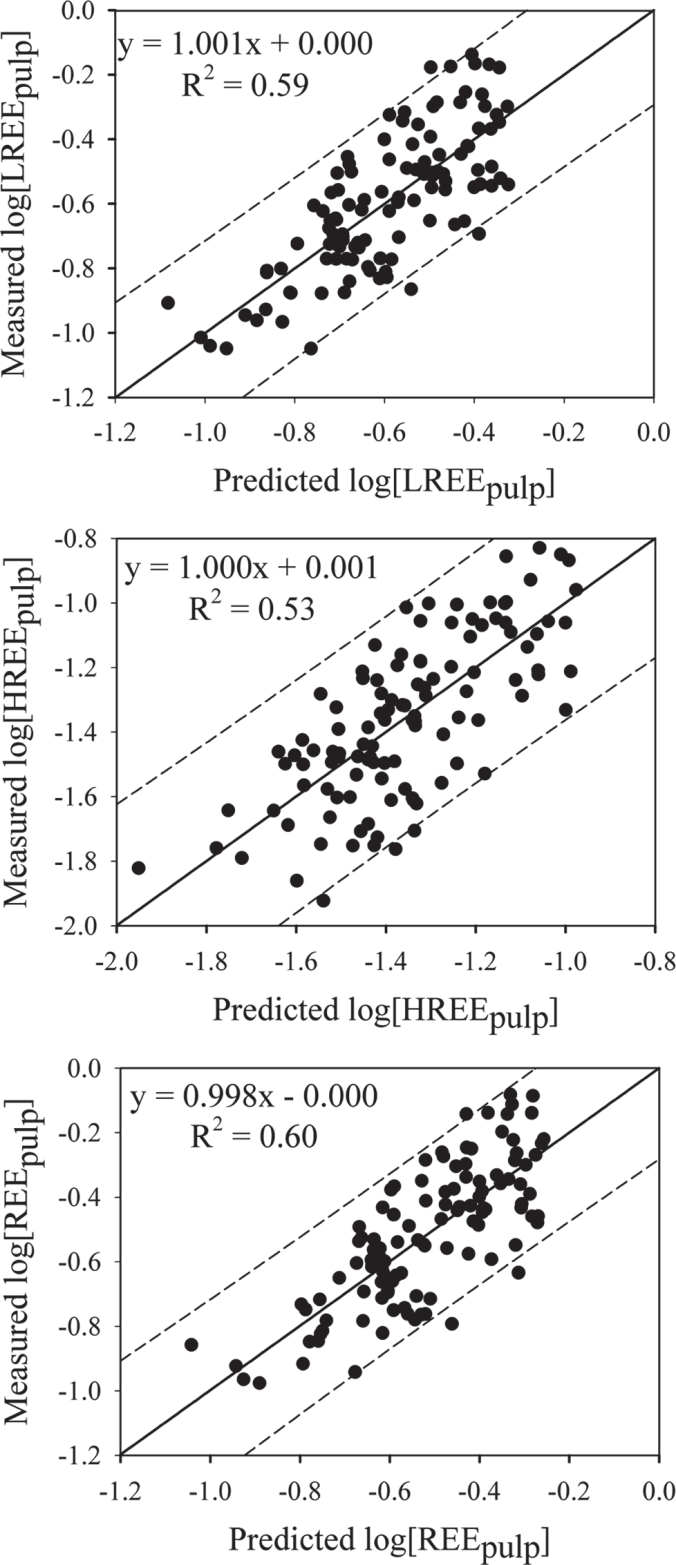
Relationship between the measured log[REE_pulp_] and the predicted log[REE_soil_].

The relationship between the measured log[REE_pulp_] and the corresponding predicted log[REE_pulp_] is shown in Fig. 4. Most of the predictions for log[LREE_pulp_], log[HREE_pulp_] and log[REE_pulp_] were within the 95% prediction intervals, indicating that the prediction models derived in the present study display good accuracy. Moreover, the root-mean-square error (RMSE) values were 0.14, 0.18 and 0.14 for the LREE, HREE and REE prediction models, respectively (Table 4). Therefore, these prediction equations were reliable predictors of REE concentrations in the navel orange pulp, and the equation for the total REE in the pulp provided the highest predictability (RMSE = 0.14).

### Correlation between soil REE and internal fruit quality of navel oranges

The contents of TA, TSS and Vc in the navel orange pulp ranged from 0.4% to 0.9%, 10.2% to 16.3% and 31.1 to 69.2 mg 100 g^-1^, with averages of 0.60%, 12.8% and 47.1 mg 100 g^-1^, respectively. A correlation analysis was conducted between navel orange quality and REE in the soil (Table 5). Significant positive relationships between the TA, TSS, Vc, LREE, and HREE in the soil were observed, indicating that soil LREE and HREE are beneficial for forming and increasing the flavor and nutritional value of navel oranges and thus to improving navel orange internal quality. Moreover, total soil REE correlated strongly with TA (*r* = 0.52, P < 0.01), TSS (*r* = 0.48, P < 0.01) and Vc (*r* = 0.56, P < 0.01), indicating that navel orange internal quality improves with higher total REE present in the soil in the investigated area.

**Table 5:** Correlation coefficients (*r*) among internal fruit quality indicators and soil REE.

	TA^a^	TSS^b^	Vc^c^
La	0.49**	0.46**	0.50**
Ce	0.43**	0.37**	0.46**
Pr	0.49**	0.48**	0.50**
Nd	0.49**	0.49**	0.50**
Sm	0.48**	0.50**	0.52**
Eu	0.43**	0.51**	0.49**
Gd	0.45**	0.37**	0.46**
Tb	0.42**	0.36**	0.46**
Dy	0.39**	0.36**	0.46**
Ho	0.37**	0.36**	0.46**
Er	0.37**	0.36**	0.46**
Tm	0.37**	0.37**	0.46**
Yb	0.38**	0.37**	0.46**
Lu	0.38**	0.37**	0.46**
Y	0.35**	0.34**	0.44**
LREE	0.52**	0.49**	0.55**
HREE	0.38**	0.35**	0.45**
ΣREE	0.52**	0.48**	0.56**

^a^ Titratable acidity

^b^ Total soluble solids

^c^ Vitamin C

** Significant at P < 0.01.

## Discussion

Generally, the content of REE in the main organs of the navel orange decreased in the following order: root > leaf > peel > pulp [7,22]. The main edible part of the navel orange is the pulp, although there are reports describing the utilization of the navel orange peel [33,34]. Therefore, from a food safety perspective, the present study focused on REE content in navel orange pulp. The REE content in navel orange pulp studied was approximately 14 times lower than the food safety limit set in China (MHPRC 2005 [32]). The REE accumulation capacity of the navel orange pulp is relatively low in our study, with an average transfer factor (TF) of 0.003; similar results were also obtained by Wang et al. (2009) with TF of 0.002 [24] and Yu et al. (2009) with TF of 0.008 [25]. In rare earth ore area of south China, some high TFs of REE were reported for the edible parts of vegetables, including potato (0.032), green vegetable (0.026), taro (0.125) and water spinach (0.050), and REE concentrations in these vegetables exceeded the food safety limit in China [26,27]. Therefore, it should be noted that planting vegetables in the rare earth ore area of south China can compromise food safety; however, the risk is reduced if navel oranges are the cultivated crop.

When comparing the TFs of LREE and HREE, most of the TF values for the HREE are higher than those for the LREE in navel orange pulp. One possible reason for this result is that although the content of the LREE in soils is markedly higher than that of the HREE, plants have the capacity to self-adjust, resulting in the translocation of only small amounts of these metals to the aerial parts of plants [2]. Another possible reason is that transition metals are transferred through the xylem from the bottom to the top of the plant largely as complexes [35]. Previous research has shown that the stability constant of the REE complexes for most organic ligands increased with increasing REE atomic number [36]. Therefore, HREE preferentially bind to the organic ligands and migrate upward with these ligands in the xylem, which may cause HREE enrichment in the aerial parts of plants.

The results of the path analysis were consistent with the stepwise multiple regression equation, including that the soil REE content, pH, CEC and Fe_ox_ were the most important factors for explaining the variability in REE content in navel orange pulp in this study. The negative effect of pH on REE content in navel orange pulp was consistent with the general expectation that REE exhibit higher bioavailability at lower soil pH values; as the soil pH increases, the REE are more likely to precipitate and less likely to be released from soils [36]. CEC is a direct reflection of the soil cation buffer capacity, and a high CEC increases the ability of the soil to retain cationic metals, reducing their uptake by plants [10]. Previous studies have indicated that soil REE may originate from the dissolution of Fe oxyhydroxides [37]. Thus, a greater Fe oxide content in the soil leads to more REE and a larger bioavailable pool of REE. Furthermore, LREE in soil more easily bind to Fe oxide [38], which explains why the regression coefficient of Fe oxide in equation 12 is markedly higher than that in equation 13. Soil OC content is generally believed to be a critical factor influencing the bioavailability of soil metals. By contrast, significant correlations between OC content and REE content of navel orange pulp were not observed in the present study. Some previous studies have also confirmed that soil OC content has no significant effect on the bioavailable content of soil REE [10,39]. One possible reason may be that REE combined with soil organic matter are not easily released and are thus unlikely to be taken up by plants.

The prediction models developed in this study can be used to obtain reliable predictions of REE contents in navel orange pulp and therefore to assess the potential risk to humans. In addition, these models can also assist in the management of navel orange orchard soils to ensure the safety of fruits. For example, according to the prediction model, when cultivated on soil with high REE content, pulp REE content can be decreased by increasing soil pH. In this study, the ranges of REE content, pH, CEC and Fe_ox_ are sufficiently wide to represent the soil properties and soil REE contents in the navel orange production location of Xinfeng. Back-calculation from these models may not be accurate for soils in which the common variables (soil REE content and pulp REE content, pH, CEC and Fe_ox_) are outside the boundaries for which these models were derived [14]. Because the food safety limit of REE content in fruit is outside the boundary of equation 14, an accurate threshold value for soil REE in Xinfeng County cannot be obtained by back-calculation.

The prediction models can also be applied to determine whether the soil is suitable or unsuitable for safe food production [18]. Therefore, based on equation 14, an extreme case was used to estimate the soil as suitable or unsuitable for safe food production under a worst-case scenario, with a minimum pH and CEC and a maximum soil REE content and Fe_ox_. In the literature, the maximum soil REE content in south China is reported to be 1038 mg kg^-1^ [24]. The minimum pH and CEC and the maximum Fe_ox_ were determined from this investigation, not from the literature, due to the large variations in these values observed in the literature. These values were determined to be 3.75, 5.08 cmol kg^-1^, and 96.4 g kg^-1^, respectively. When these values were used in equation 14, a navel orange pulp REE content of 1.13 mg kg^-1^ dry weight was calculated. When converting the REE dry-pulp-weight content into the REO fresh-pulp-weight content with a water content of 87% and a conversion factor of 0.86 [38], the total REO content in navel orange pulp was found to be approximately 0.171 mg kg^-1^ fresh weight, which is much lower than the food safety limit in China (0.7 mg kg^-1^ fresh weight) (MHPRC 2005 [29]). Therefore, even if the soil REE content reaches 1038 mg kg^-1^, the REE content in navel orange pulp will not exceed the food safety limit. This finding further confirms that there is a very low risk of producing navel oranges with REE contents exceeding the food safety limit, even when they are cultivated in an area with a high REE baseline concentration in south China.

In addition, REE in soil have positive effects on the internal quality of navel oranges, increasing both the TA and TSS contents in navel orange pulp. The levels of TA and TSS represent the contents of organic acid and sugar in fruit, respectively, and they are key components in the perception of sour and sweet [40]. Therefore, increases in the levels of TA and TSS can give the navel orange a more intense tart-sweet flavor. The content of Vc is a reflection of the nutritional value of the fruit [41]. REE in soil also increase the Vc content, resulting in improvements to the nutritional value of navel oranges. Generally, under routine methods of water and fertilization management, the cultivation of navel oranges in rare earth ore areas in south China with soil REE ranging from 38.6 to 546 mg kg^-1^ is beneficial for improving the internal fruit quality, and the REE food safety limit is not exceeded in the pulp of these navel oranges.

## Conclusions

The present results showed that soil REE content, pH, CEC, and Fe_ox_ were significant variables affecting pulp REE concentrations. The total REE contents in soils were safe for planting navel oranges in rare earth ore area of south China. Even when total soil REE content was as high as 1038 mg kg^-1^, the navel orange was still safe enough for consumption. Under routine methods of water and fertilization management, internal fruit quality of navel orange increased with the increase of soil REE in the study area.
